# Probiotic membrane vesicles: emerging tools for disease treatment

**DOI:** 10.20517/mrr.2025.20

**Published:** 2025-06-24

**Authors:** Jari Verbunt, Frank R. M. Stassen

**Affiliations:** ^1^Department of Medical Microbiology, Infectious Diseases & Infection Prevention, School of Nutrition and Translational Research in Metabolism (NUTRIM), Maastricht University Medical Center+, Maastricht 6202 AZ, the Netherlands.; ^2^Department of Human Biology, School of Nutrition and Translational Research in Metabolism (NUTRIM), Maastricht University Medical Center+, Maastricht 6229 ER, the Netherlands.

**Keywords:** Probiotics, bacterial membrane vesicles, therapeutics

## Abstract

Probiotics are widely recognized for their health benefits, particularly in disease prevention and treatment. Recent research suggests that their therapeutic effects may be linked to the bacterial membrane vesicles (bMVs) they release. These nanoscale vesicles, secreted during probiotic growth and metabolism, facilitate intercellular communication through efficient material transport and signaling. With their biocompatibility and targeting properties, probiotic bMVs hold promise for medical applications. This review examines their biogenesis, bioactive components, functions, and role in disease treatment, while also discussing future research directions to unlock their full therapeutic potential.

## INTRODUCTION

Probiotics - live microorganisms beneficial to human health - have been studied for over a century, with early evidence from Elie Metchnikoff in 1907 linking fermented food to longevity^[1]^. Research has since shown probiotics help maintain microbiota balance, support the immune system, aid digestion, and prevent diseases. Their popularity has led to widespread commercial use. Typical examples of probiotics, such as *Bifidobacterium* and *Lactobacillus*, offer various health benefits, including detoxifying pollutants, transforming mycotoxins, synthesizing essential vitamins, and fermenting fiber^[2-4]^. They help prevent diseases caused by pathogenic bacteria by enhancing intestinal barrier integrity and modulating the immune response^[5,6]^. Probiotics also support gut microbiota balance, improve nutrient bioavailability, reduce lactose intolerance, and alleviate allergies^[7,8]^.

While significant advances have been made in probiotic research, their precise molecular mechanisms of action are still not fully understood. Probiotics benefit the body through several key mechanisms. They compete with pathogens for nutrients and receptor sites, making it harder for harmful bacteria to survive. Moreover, they also produce (anti)microbial substances such as short-chain fatty acids, organic acids, hydrogen peroxide, and bacteriocins, which reduce pathogenic bacteria in the gut. Additionally, probiotics strengthen the intestinal barrier by stimulating mucin production and regulating tight junction proteins (e.g., occluding, claudin) to enhance gut integrity, thereby preventing leaky gut and systemic inflammation. Probiotics have also been shown to modulate the immune system by interacting with the gut-associated lymphoid tissue (GALT) and influencing dendritic cells, macrophages, and lymphocytes, promoting anti-inflammatory cytokine production and improving immune responses. Furthermore, probiotics contribute to gut-brain axis communication by producing neurotransmitters such as serotonin, GABA, and dopamine, which affect mood, behavior, gut motility, and stress regulation.

## BACTERIAL MEMBRANE VESICLES

More recently, the effects of probiotics have been attributed to bacterial membrane vesicles (bMVs). bMVs can be classified into outer membrane vesicles (OMVs) from Gram-negative bacteria and cytoplasmic membrane vesicles (CMVs) from Gram-positive bacteria. OMVs, first observed in 1965, originate from the bacterial outer membrane and range from 20-250 nm, while CMVs are derived from the cytoplasmic membrane and are 20-400 nm in size^[9]^. Several models explain OMV formation, including membrane blebbing, peptidoglycan accumulation, lipopolysaccharide (LPS) remodeling, and bilayer-couple effects^[10]^. However, some OMVs contain cytoplasmic components, suggesting alternative mechanisms such as explosive cell lysis or a regulated vesicle production process^[9]^. CMVs were initially overlooked due to the thick peptidoglycan layer in Gram-positive bacteria, but their existence was confirmed over 20 years ago. Their formation likely involves enzymatic degradation of peptidoglycan, leading to vesicle release through pores with the aid of prophage-encoded lysins^[11]^. Despite ongoing research, the precise mechanisms behind CMV production remain enigmatic.

Pertaining to the safety of probiotic-derived membrane vesicles, it is noted that the commensal microbiota, consisting of approximately 10^12^-10^14^ bacteria^[12]^, already produce astronomical amounts of bMVs as part of their lifecycle^[13]^. Under normal, symbiotic conditions, these vesicles contribute to the homeostasis between microbes and the host. As such, the therapeutic administration of additional bMVs warrants careful safety evaluation. The safety of probiotics-derived membrane vesicles can be assessed through toxicity studies, such as oral administration in mice or in vitro exposure of host cells. Previous studies have shown that oral administration of vesicles derived from *Limosilactobacillus reuteri*^[14]^, *Lactobacillus rhamnosus*^[15]^, and *Akkermansia muciniphila*^[16]^ was not only safe but also provided protection against dextran sulfate sodium (DSS)-induced colitis in mice. *In vitro* experiments offer further insights into the interactions between probiotic-derived membrane vesicles and specific host cells. For example, vesicles produced by *Lacticaseibacillus casei* and *Lactiplantibacillus plantarum* have been shown to reduce proinflammatory markers such as TNFα while increasing anti-inflammatory cytokines like IL10^[17,18]^. As the cargo of probiotic membrane vesicles originates from bacteria that typically do not express immunogenic toxins or potent virulence factors, these bMVs are generally considered non-pathogenic and safe for oral administration. An overview of proposed probiotic strains used for membrane vesicle production and their associated bacterial cargo is provided in Table 1.

**Table 1:** Overview of probiotic bacterial strains producing membrane vesicles and their relevant cargo discussed in this review

**Strain**	**Phylum**	**Relevant cargo**	**Suggested target disease(s)**	**Sources**
*Akkermansia muciniphila*	Verrucomicrobiota	Not reported	IBD, metabolic syndrome	[16,19,20]
*Bacteroides fragilis*	Bacteroidota	Polysaccharide A/B	Infectious disease	[21]
*Bifidobacterium longum*	Actinomycetota	Mucin-binding protein	Infectious disease	[22]
*Clostridium butyricum*	Bacillota	Not reported	IBD	[23]
*Escherichia coli* ECOR12	Pseudomonadota	Various miRNAs	Neurological disease	[24]
*Escherichia coli* Nissle 1917	Pseudomonadota	LPS (source 1) Outer membrane protein A/C (source 2)	Metabolic syndrome, Infectious disease	[25,26]
*Escherichia coli* Nissle 1917	Pseudomonadota	Various miRNAs	Neurological disease	[24]
*Lacticaseibacillus casei*	Bacillota	Various, including membrane constituents and proteases (source 2)	IBD	[17,27]
*Lacticaseibacillus paracasei*	Bacillota	Not reported	Cancer	[28]
*Lacticaseibacillus rhamnosus*	Bacillota	Not reported	Cancer	[29]
*Lactiplantibacillus plantarum*	Bacillota	Cell surface proteins (source 2) RNA, lipoproteins (source 3)	IBD	[18,30,31]
*Lactiplantibacillus plantarum*	Bacillota	Not reported	Neurological disease	[32]
*Ligilactobacillus salivarius*	Bacillota	Salivaricin B	Infectious disease	[33]
*Limosilactobacillus reuteri*	Bacillota	Lipids including lycerols and sphingolipids; proteins including risobomal subunits (source 1) Antimicrobial peptides (source 2)	IBD	[14,34]
*Propionibacterium freudenreichii*	Actinomycetota	Various proteins involved in bacterial energy metabolism	IBD	[35]

IBD: Inflammatory bowel disease; miRNAs: microRNAs; LPS: lipopolysaccharide.

## BIOACTIVE COMPONENTS OF PROBIOTIC bMVS

Proteins in or on probiotic EVs are able to bind to host cell receptors, influencing immune regulation, cell proliferation, and apoptosis. They are also found to affect metabolism and gene expression in host cells. For example, proteins in *Lactobacillus casei*-BL23 bMVs include enzymes, signaling molecules, and heat shock proteins, which protect cells and modulate immune responses^[27]^. bMVs from *Propionibacterium freudenreichii* contain SlpB, which exerts anti-inflammatory effects through the NF-κB pathway^[35]^.

Although research is limited, bMVs from *Lactobacillus plantarum* contain both DNA and RNA, which can modulate host gene expression^[36]^. MicroRNAs (miRNAs) and small RNAs (sRNAs) in these bMVs may influence cellular function similarly to those in pathogenic bacteria. For instance, sRNAs from *L. plantarum* EVs regulate Tp53 expression, suggesting potential applications in tumor treatment^[31]^. Probiotic bMVs have a phospholipid bilayer containing phospholipids, cholesterol, and other molecules that stabilize structure and enable fusion with host cells. Some lipids can regulate inflammatory signaling. For example, lipoteichoic acid in *Lacticaseibacillus rhamnosus* bMVs activates Toll-like receptor 2 (TLR2), leading to increased expression of the anti-inflammatory cytokine IL-10 in dendritic cells^[37]^. The ability of bMVs to transport TLR agonists has been described previously, with a focus on LPS. LPS - also referred to as bacterial endotoxin - is a major component of the outer membrane of Gram-negative bacteria and is commonly found on bMVs^[38,39]^. As a potent activator of TLR4, LPS induces downstream immune responses and inflammation. However, it is essential to note that different types of LPS carried by bMVs can exert different immunological effects. For example, LPS carried by vesicles of *E. coli* Nissle 1917 has been shown to elicit more anti-inflammatory effects on Caco-2 cells compared to LPS from ECOR 12 *E. coli* vesicles^[25]^. Specific acylation patterns of LPS may underlie these differences, as hexa-acylated LPS has been reported to be a significantly more potent activator of TLR4 than tetra-/penta-acylated counterparts^[40]^. These distinctions are particularly relevant when selecting probiotic strains for vesicle production. As for Gram-positive bioactive components of bMVs, there have been studies on TLR2 agonists such as peptidoglycan and teichoic acids such as LTA (lipoteichoic acid)^[41]^. These TLR agonists are known to trigger host inflammatory responses^[42]^. Less commonly described bioactive components of bMVs include TLR5 agonists like flagellin^[43]^, as well as TLR7/8 and TLR9 agonists such as bacterial RNA and DNA^[44]^. A substantial body of literature exists on the various bioactive substances characterizing bMVs and the factors that influence their composition^[45]^. In the context of probiotics, bMVs have emerged as promising therapeutic biomaterials due to their immunomodulatory properties mediated by their bioactive cargo. The application of probiotic-derived bMVs, therefore, holds considerable potential for treating various conditions, including - but not limited to - inflammatory bowel disease (IBD), neurological disorders, metabolic syndrome, and cancer. In this work, we elaborate on empirical findings and molecular mechanisms supporting the therapeutic potential of probiotic-derived membrane vesicles in mitigating these diseases.

## IBD

IBD, which includes ulcerative colitis (UC) and Crohn’s disease (CD), is characterized by chronic inflammation of the intestines, resulting in symptoms such as diarrhea, abdominal pain, and weight loss. Its development is multifactorial and commonly involves immune system dysfunction with abnormal cytokine production (such as TNF-α, IL-1β, IL-6, and IL-10), and changes in the expression of tight junction proteins (including Claudin, ZO-1, and JAM-A). These factors contribute to disruptions in the gut microbiota and damage to the intestinal mucosal barrier in a context-dependent manner^[46,47]^.

**Figure 1:**
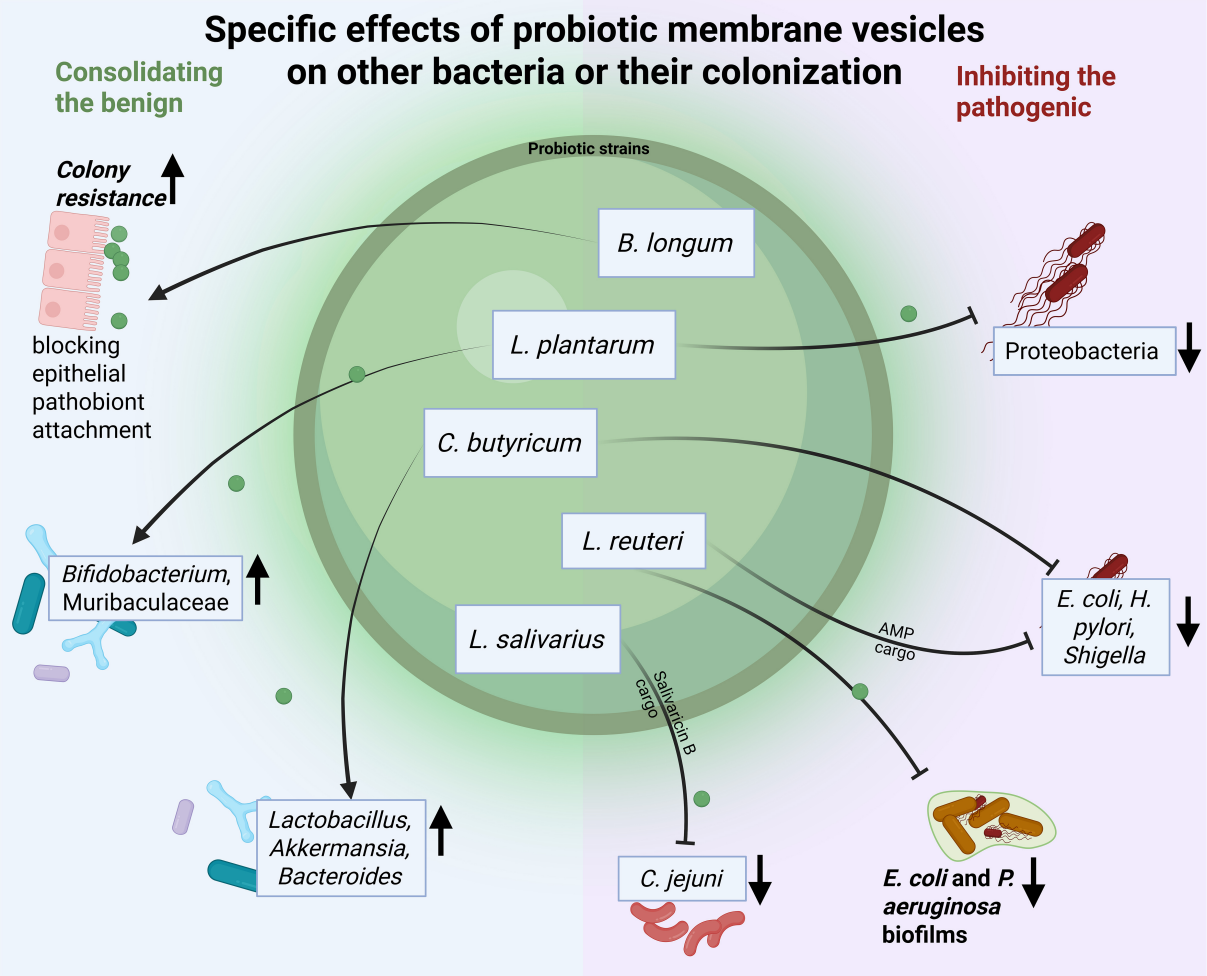
Probiotic membrane vesicles and their described interactions with gut bacteria and their colonization. Vesicles produced by probiotic strains have been found to stimulate colonization by other symbiotic gut bacteria while inhibiting the outgrowth of pathobionts. Created with BioRender.com. AMP: Antimicrobial peptide.

The rising incidence of IBD, influenced by lifestyle and environmental factors, significantly impacts health and quality of life^[46]^. Recently, probiotic extracellular vesicles have gained attention for their anti-inflammatory and immunomodulatory potential, emerging as promising treatments for inflammatory diseases. Individuals with IBD typically exhibit a reduced abundance of bacteria that are inversely associated with inflammation while displaying an increased abundance of bacteria that are directly linked to inflammation^[47]^. Probiotic-derived bMVs have been shown to either inhibit the growth of inflammation-related pathogens or to facilitate the proliferation of anti-inflammatory bacteria [Figure 1]^[23,30]^. These findings have been validated *in vivo*; administration of *Lactobacillus plantarum*-derived CMVs has been found to reduce Pseudomonadata (pro-inflammation) and increase *Bifidobacteria* and Muribaculaceae (anti-inflammation) in DSS-induced colitis in mice^[30]^. Independently, it was found that the DSS-induced progression of experimental colitis could be halted through oral administration of *Akkermansia muciniphila* bMVs^[16]^. Moreover, it was found that after ingesting bMVs derived from *Clostridium butyricum*, the relative abundance of pathogenic bacteria such as *Helicobacter pylori* and *Shigella* decreased, while at the same time, a marked increase in the relative abundance of *Lactobacillus*, *Akkermansia*, and *Bacteroides* was observed^[23]^. These findings underscore the potential of probiotics-derived bMV as a novel therapeutic strategy to restore dysbiosis of the gut microbiome in IBD.

## METABOLIC DISEASE

Metabolic syndrome denotes a group of disorders affecting energy metabolism and nutritional balance, including diabetes, obesity, and lipid metabolism dysfunctions. These conditions are often characterized by insulin resistance, elevated blood sugar, abnormal lipid levels, hypertension, and inflammation in adipose tissue. Their development is influenced by genetic predisposition, unhealthy lifestyle choices - such as high-sugar, high-fat diets and physical inactivity - as well as environmental factors and insulin resistance^[48,49]^. In the context of gut microbiota dysbiosis, Choi *et al.* revealed the implication of gut microbiota–derived EVs in the worsening of diet-induced metabolic disorders. They demonstrated that the detrimental effect of a high-fat diet resulted in dysbiosis of the gut microbiome and disturbance of the glucose metabolism mediated by *Pseudomonas panacis* LPS-containing bMVs^[50]^. This suggests that gut microbiota dysbiosis could contribute to the onset and progression of metabolic diseases such as obesity and diabetes. Moreover, the role of bMV production by endogenous microbiota is increasingly studied in the context of metabolic syndrome^[13]^.

In contrast, probiotic bMVs have been shown to have a positive effect in regulating metabolic disorders and alleviating diabetes. For example, bMVs produced by the probiotic *E. coli* strain Nissle 1917 reduced body weight, decreased blood glucose, and increased plasma insulin levels in obese mice^[26]^. Likewise, oral administration of *Akkermansia muciniphila* EVs to mice on a high-fat diet decreased gut barrier permeability, reduced body weight gain and liver inflammation, improved glucose tolerance, and increased the expression of genes involved in lipid metabolism and homeostasis^[19,20]^. These changes also improved the microbial composition, shifting the gut microbiota profile closer to that of the controls.

## CANCER

Cancer is a disease marked by the uncontrolled growth and spread of abnormal cells. Its key characteristics include invasiveness, metastasis, histological abnormalities, and clinical symptoms such as weight loss and fatigue^[51]^. The underlying mechanisms involve genetic mutations that drive excessive cell proliferation, complex interactions within the tumor microenvironment that support cancer cell survival and immune evasion, and the dysregulated activity of immune cells and cytokines. These factors collectively influence cancer progression and impact treatment outcomes, making cancer a major threat to human health. bMVs from harmful bacteria can contribute to tumor development by promoting inflammation, altering immune responses (e.g., by inducing immune exhaustion), or even by directly affecting the DNA of host cells. For instance, vesicles from *Helicobacter pylori* - a bacterium strongly linked to gastric cancer - can deliver virulence factors that damage epithelial cells and create an environment conducive to cancer progression^[52,53]^. These vesicles can manipulate host signaling pathways, suppress immune surveillance, and enhance angiogenesis, all of which support tumor growth. Additionally, Tyrer *et al.* demonstrated that *E. coli* bMVs could be internalized by Caco-2 cell lines, inducing DNA double-strand breaks and aneuploid replication of cells, and revealed the genotoxicity of these bMVs to host cells, suggesting their potential carcinogenicity^[54]^. On the other hand, bMVs derived from probiotic bacteria - such as *Lactobacillus* and *Bifidobacterium* - appear to offer a protective counterbalance. Studies have shown that bMVs from *Lacticaseibacillus paracasei* can inhibit tumor growth and spread, while also promoting cancer cell death by downregulating Bcl-2 expression^[28]^. Likewise, extracellular vesicles from *Lacticaseibacillus rhamnosus* have been shown to reduce Bcl-2 expression in HepG2 liver cancer cells, triggering apoptosis and demonstrating cytotoxic effects^[29]^. These beneficial vesicles can reinforce the gut barrier, reduce inflammation, and modulate immune responses in ways that discourage cancer development. Some even contain molecules that directly target tumor cells, triggering processes like apoptosis (programmed cell death) or blocking pathways that cancer cells rely on to grow and survive. Moreover, bMV can influence the tumor microenvironment - the ecosystem of cells and molecules that surrounds and supports cancer. Probiotic-derived bMVs may help “re-educate” immune cells within this environment, encouraging them to recognize and attack tumor cells rather than ignoring them. Early research suggests that the genetic material carried within these vesicles - such as miRNAs and sRNAs - can enter human cells and subtly shift the way genes are expressed, potentially silencing cancer-promoting genes or activating protective ones^[55]^.

In summary, bMVs represent a new frontier in cancer research, revealing how even the smallest components of the microbiome can have outsized effects on human health and disease. For example, engineered vesicles from probiotics might one day be used to deliver drugs or gene therapies directly to tumors, offering a precise and less toxic alternative to traditional treatments^[56]^.

## NEUROLOGICAL DISEASE

Neurological disorders, encompassing neurodegenerative and neuroinflammatory pathologies, exert a profound influence on quality of life. Their pathogenesis is characterized by neuronal damage, dysregulation of neurotransmitter homeostasis, neuroinflammatory cascades, and structural modifications within the nervous system. These processes are modulated by a complex interplay of genetic predispositions and environmental factors, including aberrant protein expression (e.g., CD209), inflammatory mediators (e.g., iNOS, Arg1), and neurotrophic signaling molecules^[57]^.

Emerging evidence posits that bMVs derived from probiotics possess the capacity to interact with, and potentially traverse, the blood-brain barrier^[58]^. This interaction facilitates their modulation of central nervous system dynamics, notably through the regulation of neuroinflammatory responses, thereby influencing the pathophysiology of disorders such as Alzheimer’s disease and major depressive disorder^[59]^. Furthermore, the inherent stability and bioactivity of probiotic-derived bMVs position them as prospective vectors for targeted pharmacotherapeutic delivery within the CNS.

These vesicles encapsulate an array of neuroprotective constituents, including antioxidant compounds and nerve growth factors, which collectively mitigate neuronal apoptosis and foster cellular repair mechanisms^[60]^. Specific probiotic bMVs have demonstrated the ability to enhance stem cell proliferation and synaptogenesis, thereby promoting neural regeneration. For instance, bMVs originating from *Escherichia coli* strains ECOR12 and EcN have been shown to induce dendritic cell maturation, evidenced by upregulation of CD83 and concomitant downregulation of CD209 expression^[24]^. Moreover, probiotic bMVs exhibit pronounced anti-inflammatory properties within the central nervous system. bMVs derived from the Lactobacillaceae family attenuate LPS-induced microglial activation by modulating the expression of inflammatory markers, notably upregulating Arg1 (an M2 phenotype marker) while suppressing iNOS (an M1 phenotype marker)^[59]^. Additionally, bMVs from *Lactiplantibacillus plantarum* have been observed to augment the expression of brain-derived neurotrophic factor (BDNF) in hippocampal neurons, eliciting antidepressant-like effects in preclinical models^[32,61,62]^. These findings underscore the therapeutic potential of probiotic EVs in ameliorating neuroinflammatory and neurodegenerative conditions^[63]^.

## INFECTIOUS DISEASE

With respect to infectious disease, probiotics-derived bMVs could confer beneficial effects on host immunity and pathogen resistance. Firstly, probiotic bacteria have been shown to maintain mucosal layers and gut-barrier function through vesicle release. Vesicles released by *B. fragilis*^[21]^ and *E. coli* Nissle 1917^[26]^ can locally downregulate proinflammatory signaling detrimental to gut-mucosal homeostasis in the context of infectious disease. Moreover, some probiotic-derived bMVs could directly upregulate tight junction proteins, thereby contributing to reduced permeability and translocation of pathogenic bacteria and their virulence factors^[64]^. Such examples illustrate how probiotics might consolidate gut-barrier function through the release of bMVs, thereby mitigating infectious disease mechanisms. *Bifidobacterium longum* bMVs have been found to facilitate the attachment of parent bacteria to epithelial cell surfaces, potentially preventing colonization by pathobionts^[22]^ [Figure 1].

bMVs produced by probiotics might also directly interact with the host’s immune system relevant in the context of infectious diseases, either by training the innate immune system to recognize microbe-associated molecular patterns (MAMPs) or by regulating (adaptive) immune responses that might otherwise cause tissue damage. *L. rhamnosus* bMVs were found to be potent immunomodulatory agents through stimulation of pathogen recognition receptor TLR2 in monocytes, priming these host cells to respond more effectively to the presence of infectious agents^[65]^. By carrying polysaccharide A, a common MAMP, *B. fragilis* bMVs can activate TLR2 on dendritic cells. This activation primes the host immune cells to respond more effectively to inflammatory stimuli while retaining a tolerogenic phenotype in their absence. Mice pre-treated with these *B. fragilis* bMVs indeed exhibited enhanced resistance to colitis and systemic infections^[21]^. In addition, bMVs released by *B. fragilis* and *L. plantarum* could directly induce anti-inflammatory cytokine release (IL-10, TGF-β) by regulatory T cells, thereby reducing excessive inflammation in infectious disease^[21]^.

Besides such mechanisms involving the host immune system, bMVs from probiotic bacteria could also directly affect other bacteria and inhibit their growth by transferring antimicrobial peptides and proteases. *In vitro* studies have shown that *L. reuteri* bMVs endogenously containing antimicrobial peptides can inhibit the growth of *E. coli*^[34]^. For example, *Lactobacillus salivarius* produces Salivaricin B, a cyclic antibiotic, and its membrane vesicles exhibit antimicrobial properties against *Campylobacter jejuni*, a common enteropathogen^[33]^. Additionally, bMVs can interfere with quorum sensing in competing bacteria; for instance, vesicles from *Limosilactobacillus reuteri* have been shown to disrupt biofilm formation by potential pathogens such as *E.coli* and *P. aeruginosa*^[66]^.

The ability of probiotic-derived bMVs to (i) reinforce epithelial/mucosal barriers; (ii) modulate immune responses; and (iii) exert direct antimicrobial effects on other (pathogenic) bacteria renders them promising candidates in the prevention and treatment of infectious diseases. Although preclinical evidence is accumulating, clinical translation remains limited.

## CONCLUSION

Probiotic-bMVs are increasingly recognized for their pivotal role in facilitating intercellular communication, thereby exerting significant influence on both physiological and pathological processes. Advances in this domain are anticipated to enhance our comprehension of cellular and molecular biology, while concurrently paving the way for innovative approaches to disease diagnosis and therapeutic intervention. The investigation of probiotic bMVs not only enriches the scientific understanding of biological systems but also catalyzes innovation in clinical applications. As a novel platform for the delivery of bioactive molecules, these vesicles exhibit considerable potential across diverse fields, including drug delivery, diagnostics, tissue engineering, and immunotherapy. As their biogenesis and factors that influence their nature and properties are currently being elucidated, bioreactor-scale production of inherently safe probiotics and/or their bMVs will be scalable and possible in the near future.

Within the realms of medicine and biotechnology, probiotic bMVs demonstrate substantial promise. Their multifaceted applications - spanning personalized medicine, oncological therapies, management of neurological disorders, and regenerative medicine - position them as integral components of future clinical strategies. Furthermore, probiotic bMVs constitute a novel paradigm for intercellular signaling and molecular exchange. As research advances, considerable health benefits could be attained through their interactions with the host, particularly given prior evidence of systemic bMV dissemination to specific target tissues^[67]^. Enhancing the targeting and homing mechanisms of bMVs and their cargo could further improve their therapeutic efficacy in disease treatment.

As research into their mechanisms and applications proliferates across the disciplines of medicine, biotechnology, and the broader life sciences, these vesicles are expected to contribute significantly to the advancement of human health and the progression of scientific discovery.

## DECLARATIONS

### Copyright

© The Author(s) 2025.
